# Addressing class imbalance in traumatic brain injury prognostication: A survey of resampling approaches

**DOI:** 10.3934/Neuroscience.2026008

**Published:** 2026-03-25

**Authors:** Nor Safira Elaina Mohd Noor

**Affiliations:** 1 Department of Neurosciences, School of Medical Sciences, Health Campus, Universiti Sains Malaysia, 16150 Kubang Kerian, Kelantan, Malaysia; 2 Brain Behaviour Cluster, School of Medical Sciences, Health Campus, Universiti Sains Malaysia, 16150 Kubang Kerian, Kelantan, Malaysia

**Keywords:** resampling, electroencephalogram, generative adversarial network, traumatic brain injury, imbalance

## Abstract

In recent years, the classification of electroencephalogram (EEG) signals has attracted considerable interest, particularly in clinical and cognitive neuroscience applications. However, the problem of class imbalance often undermines the performance of classification algorithms and leads to biased predictions in favor of the majority class. In this paper, I provide a review of data approaches that address the class imbalance problem in EEG classification. I explored methods such as oversampling, undersampling, and the application of advanced algorithms such as Generative Adversarial Networks (GANs), which generally showed better overall performance in dealing with class imbalance. In this paper, I reviewed work published in the last 5 years to evaluate the current state of the art in imbalance. In addition, I provide insight into potential gaps in EEG classification, especially in traumatic brain injury (TBI), and discuss the potential of data augmentation based on deep learning, i.e., GANs, which have shown promise in generating synthetic data to improve minority class representation in other application domains for the EEG-based classification model. By highlighting gaps in existing EEG classification methods and proposing GANs as a solution to class imbalance, I aim to contribute to the development of more robust prediction models that may lead to improved patient outcomes in TBI cases.

## 1. Introduction

The heterogeneous characteristics of traumatic brain injury (TBI) represent a significant health burden worldwide and require sophisticated approaches to prognosis and intervention. The use of machine learning (ML) methods has proven to be a revolutionary strategy in the clinical setting, offering significant opportunities for predicting patient outcomes. The prognosis has been determined through initial clinical evaluations, including demographic information, Glasgow Coma Scale (GCS) scores, admission CT scan findings, systemic factors such as early hypotension and increased intracranial pressure (ICP), and the type of lesion, specifically acute subdural hematoma and midline shift [1]–[3]. Electroencephalography (EEG), a non-invasive neurophysiological tool with high temporal resolution, provides direct insight into brain function, making it increasingly valuable for the monitoring and prognosis of neurological disorders, including TBI [4]. TBI is frequently marked by intricate pathophysiological mechanisms and may lead to electrophysiological irregularities detectable via EEG. Ianof and Anghinah [5] underscore the importance of EEG findings in TBI, especially in detecting brain injuries that exhibit modest electrophysiological changes, particularly in mild TBI (mTBI). This highlights the significance of EEG in therapeutic environments, since it enables the identification of abnormalities that may be easily missed.

Research has consistently shown that distinct EEG patterns following TBI offer accurate predictions. A systematic review analysis of EEG patterns has been put forward by Mohd Noor and Ibrahim [6]. A set of EEG features has been identified that enables patients to discriminate in a poor and good prognosis. These features quantify EEG features as predictors for predicting TBI outcomes such as relative EEG power, connectivity features, and spectral features, including entropy. Among these, relative power is frequently highlighted. Moreover, an increase in theta and delta power, in contrast to less significant alpha and beta activities, tends to correlate with poorer prognostic outcomes [7],[8]. A flat EEG signal, indicative of non-reactive states, is generally associated with poor prognosis, particularly in patients classified as brain dead [5],[9]. This aligns with findings that EEG power can reflect fundamental mechanisms of consciousness and potential for recovery. In contrast, reactive EEG patterns, characterized by increased electrical activity in response to stimuli, have been associated with improved outcomes in severe TBI. Additionally, studies have noted slowing of the posterior dominant rhythm [10] and diffused theta activity following TBI, which can persist and provide information about the patient's recovery. These patterns are crucial as they characterize cortical functioning and network connectivity, which can directly influence outcomes [11],[12]. Therefore, this raises the question of how advanced EEG analysis techniques can be applied to extract informative predictors that facilitate clinical diagnostics and reliable outcome predictions.

In TBI, understanding timing of outcomes and their types is crucial for effective prognostication and treatment planning. The acute phase following TBI, the first seven days post-injury, is crucial for assessing immediate neurological responses. Early post-traumatic seizures (PTSs) are frequently observed and necessitate urgent management to mitigate the potential secondary injuries [3]. The relationship between early acute seizures and outcomes remains complex, where continuous EEG monitoring indicates a high incidence of convulsive and non-convulsive seizures, demonstrating that acute seizures are prolific yet often transient. In contrast to acute seizures, posttraumatic epilepsy (PTE) develops as a recurrent seizure disorder, typically manifesting after the first week post-TBI. The impact of PTE is significant, since individuals may experience elevated rates of disability, including difficulties in everyday functioning and a higher likelihood for mental health disorders. Moreover, research indicates that individuals who acquire PTE following TBI exhibit alterations in cerebral metabolism that exacerbate results over time [13]. Mortality rates associated with PTE indicate a poor prognosis, with early seizures identified as key predictors of long-term mortality in certain populations. Zelig et al. [14] note that the timing and severity of electrical abnormalities detected on EEG in the first days after injury are significant predictors of later unfavorable outcomes, including mortality.

Advances in ML and deep learning (DL) have driven efforts to develop automated EEG-based prognostic models for TBI promising initial results [15],[16]. Conventional ML models, such as support vector machines (SVM) and random forests (RF), rely on handcrafted EEG features and provide interpretable decision rules. DL approaches, including convolutional neural networks (CNN), enable automated extraction of spatial and spectral patterns from multichannel EEG signals. Recurrent architectures, such as long short-term memory networks (LSTM), are well suited for modeling the temporal dynamics of EEG activity. Hybrid models combine multiple architectures to capture complementary information across time, frequency, and spatial domains. Despite these advances, a major challenge in developing robust and generalizable EEG-based prognostic models for TBI is class imbalance within clinical datasets. Imbalance between classes can severely compromise the effectiveness of ML models, particularly in clinical contexts where certain outcomes (e.g., severe injuries) may be significantly underrepresented. Various strategies have been proposed to counteract the detrimental effects of class imbalance. Among these, data augmentation has emerged as a promising technique to synthetically augment the available data for underrepresented classes and thereby improve the robustness and generalization of the model.

Data augmentation techniques can alleviate this problem by synthetically generating additional training samples from the minority class while retaining the inherent features of the original data. Despite being well-established in computer vision, data augmentation is under-explored for EEG data. The motivation behind the data augmentation is to make the model more robust to noise in EEG recordings. The inherent noise in EEG recordings poses a major challenge, especially with regard to the limited signal-to-noise ratio (SNR) [17]. Noise originates from various sources, including physiological factors, external disturbances, and the mechanics of the EEG systems. These interferences result in a low SNR, complicating the extraction of significant brain signals without sophisticated noise suppression methods. Given that the power of EEG signals diminishes with frequency, augmentations that maintain power band ratios at lower frequencies are especially beneficial. The lower frequency bands, including delta, theta, alpha, and beta, are essential for many EEG decoding tasks, particularly in predicting outcomes in TBI. Preserving these power band ratios guarantees that the critical physiological data embedded in these frequency bands remain unaltered throughout augmentation, enabling the model to concentrate on relevant neural features rather than extraneous noise.

The complexity of electroencephalography (EEG) data poses numerous challenges for ML, especially when using deep learning methods to categorize different EEG patterns. One critical problem is class imbalance, where certain categories (e.g., those representing certain neurological disorders) have significantly fewer instances than the predominant categories. Class imbalance can lead classifiers to favor the more dominant class labels, limiting the model's ability to recognize important but rare EEG patterns. Therefore, resampling techniques that include oversampling and undersampling methods have been implemented to mitigate these problems. In addition, Generative Adversarial Networks (GANs) have proven to be a sophisticated tool for generating synthetic data to equalize the class distribution in EEG datasets, serving as a crucial method for improving representation and facilitating more effective learning by classifiers [18].

The sampling methods, which account for 17% of the total techniques [18], can be divided into oversampling of minority groups and undersampling of majority classes. Oversampling strategies involve replicating data from minority classes to improve their representation, while undersampling involves randomly eliminating samples from the majority class to mitigate imbalance [19]. Sánchez-Gutiérrez and González-Pérez (2023) advocate exploring GANs as a viable alternative and emphasize the need for methods that address the optimization deficiencies of conventional classifiers for imbalanced datasets. The use of GANs, which account for 21% of the literature, represents an innovative method for data augmentation in EEG datasets [18]. This adversarial method improves sample quality and enables the generation of highly accurate signals that accurately reflect the underlying data distribution of minority groups. Numerous studies have shown that GANs can significantly improve the classification efficiency of minority classes, especially when integrated with additional augmentation methods [20]. Hartmann et al. [21] showed that GANs and resampling algorithms generally produce larger augmentation factors and improved accuracy metrics before and after augmentation when evaluating the effectiveness of data augmentation methods. Comparative performance highlights the need for customized augmentation methods that consider the specifics of the EEG dataset and the complexity of the task.

In this paper, I provide a comprehensive review of resampling techniques that aim to improve classifier performance by enabling effective learning from a balanced representation of minority and majority classes, thus improving sensitivity and accuracy in critical EEG classification tasks from a data-level perspective. I provide a comprehensive overview of resampling methods, focusing on oversampling, undersampling, and GANs. This analysis also includes the application of these methods in recent studies for EEG classification tasks to improve model performance. Finally, I emphasize the difficulties associated with processing imbalanced EEG datasets for predicting TBI outcomes and show how GANs can serve as effective resampling methods through deep learning, an increasingly relevant approach in ML research.

## 2. Electroencephalographic data

Electroencephalogram (EEG) recordings are acquired by positioning electrodes on the scalp to monitor brain wave activity during a specified period. EEG segments are typically evaluated for durations ranging from seconds to hours, depending on the clinical situation or study design. Continuous EEG monitoring, especially in critically ill patients [22], can last from 24 hours to several days, enabling comprehensive analysis. EEG uses multiple channels, with the number of electrodes placed over different scalp regions affecting the amount of data collected. Standard clinical EEG setups typically use 21 or 32 channels, commonly referenced with the 10–20 system, while more advanced systems may include 64, 128, or more electrodes. EEG signals are digitized and converted into numerical data, with time represented on the X-axis and the voltage amplitude of brain activity on the Y-axis for each channel. Each channel corresponds to a specific scalp electrode, capturing localized electrical fluctuations of neuronal activity. Together, these recordings form a multidimensional dataset that can be mathematically represented as:

$$X\in {\mathbb{R}}^{NMK}$$ (1)

where *N* denotes the number of EEG samples or epochs, *M* is the number of channels, and *K* is the number of time points per epoch. Each element *x_n,m,k_* represents the voltage amplitude at the *t* for channel *m* in sample *n*. This multidimensional structure preserves spatial (across channels) and temporal (over time) information, which is crucial for EEG signal processing.

EEG data consists of continuous recordings of electrical activity in the brain, segmented into smaller time intervals called epochs or segments. In EEG datasets, especially those focused on event detection such as seizure detection, significant class imbalance can occur. For instance, if only a small number of epochs contain seizure events (the minority class) compared to those where the individual is not experiencing a seizure (the majority class), the dataset becomes imbalanced. The use of resampling methods, class weighting, and advanced ML algorithms can effectively address these imbalances, thereby improving the accuracy and reliability of predictions in clinical practice [23],[24].

## 3. EEG resampling data approaches

### 3.1. Random oversampling

**Figure 1.**
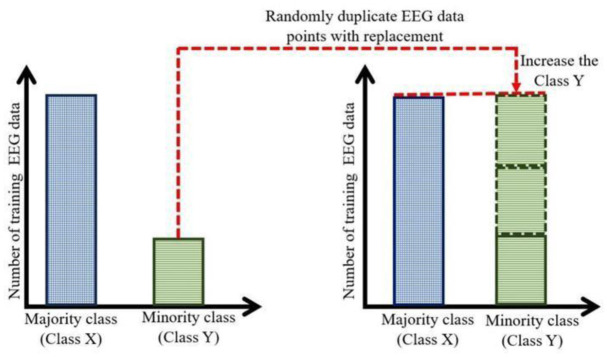
Random oversampling process of imbalanced EEG dataset.

Oversampling approaches balance a data set by creating samples or adding more samples to minority classes (i.e., creating new data) until a desired target class ratio is reached. Random oversampling involves the random replication and substitution of minority class data points until the distribution of the two classes becomes balanced. Put simply, instances with a minority class in the target variable are duplicated at random until their quantity matches that of the majority class instances (see Figure 1). Random oversampling serves as the fundamental approach that underlies various oversampling techniques, including the synthetics minority oversampling technique (SMOTE). It enhances the overall precision of a prediction model by offering researchers a greater number of samples from minority classes, mitigating the issue of class imbalance. Nevertheless, the duplicated samples resulting from random oversampling may possess identical values, hence augmenting the likelihood of overfitting. Furthermore, the utilization of random oversampling leads to an increase in the training duration of the predictive model, particularly when the dataset expands in size due to the inclusion of additional minority samples [25]. The SMOTE algorithm generates synthetic data by leveraging feature space similarities among existing minority instances. Unlike random oversampling, which duplicates existing minority class instances and may lead to overfitting, SMOTE creates new synthetic samples by interpolating between current minority class instances and their nearest neighbors.

The SMOTE algorithm operates in the following phases: First, it identifies the k-nearest neighbors for each instance in the minority class. Synthetic examples are then created by calculating the difference between the feature vector of the minority instance and that of its nearest neighbor, multiplying this difference by a random value between 0 and 1, and adding the result to the original feature vector. The simplest method for increasing the size of minority classes is standard SMOTE and its variants, including borderline SMOTE, support vector machine (SVM)-SMOTE, Logistic Regression (LR) SMOTE, and k-nearest neighbor (kNN)- SMOTE [26]–[28].

Borderline-SMOTE represents a significant refinement of the original algorithm by focusing synthetic sample generation on minority class instances near the decision boundary between classes. Xie et al. [29] demonstrated how the borderline-SMOTE improved the SMOTE algorithm by targeting specific regions of the feature space, reasoning that instances near the boundary are most susceptible to misclassification and critical for improving classifier performance. LR-SMOTE has conceptual parallels with Borderline-SMOTE, as both prioritize creating synthetic samples near the decision boundary. Mujahid et al. [30] analyzed several oversampling algorithms, highlighting that these methods address the problem of imbalanced datasets by intentionally focusing on specific areas of the feature space. Borderline-SMOTE identifies boundary instances by analyzing the proportion of majority class neighbors, while LR-SMOTE uses LR probability estimates to pinpoint key cases.

The use of oversampling methods, such as SMOTE and its variants, can enhance classification accuracy by improving the representation of minority classes in these datasets [23],[31]. In comparative analysis of resampling techniques applied to EEG data related to focal epilepsy, Varotto et al. [23] emphasized the need to use appropriate data preprocessing methods to improve minority class. The study involved analyzing stereo-electroencephalography (SEEG) signals from individuals with drug resistant focal epilepsy. Non-physiological artifacts were removed, and 3-minute continuous interictal SEEG signals recorded during awake states were selected. The signals were then divided into 36 nonoverlapping epochs of 5 seconds each, and a frequency band of 1–80 Hz was used for analysis. To address class imbalance, the researchers compared five oversampling and five undersampling procedures, as well as six ensemble methods designed for imbalanced domains. Based on the findings, the authors found that Random Undersampling (RUS) demonstrated the best performance among undersampling approaches, despite its simplicity, and was more robust across classifiers than oversampling methods. RUS, along with methods such as undersampling based on clustering (SBC) and Tomek's modification of Condensed Nearest Neighbor (CNNTL), significantly improved performance metrics such as geometric mean and balanced accuracy compared to the original dataset. Adaptive Synthetic Sampling (ADASYN) showed the best overall performance among oversampling techniques and was more sensitive to the type of classification method used.

Oversampling generally improves classification performance, particularly in terms of sensitivity. Their results suggest that the use of oversampling significantly improves the effectiveness of ML classifiers in discriminating epileptogenic and non-epileptogenic regions in EEG recordings. This view was confirmed by Zhou et al. [32], who examined the shortcomings of conventional ML methods that neglect imbalances between classes, which adversely affect patient outcomes, especially in the epilepsy classification [32]. Research shows that emphasizing the correlations between data samples during the oversampling process can significantly improve the robustness of the model. Therefore, it is essential to integrate comprehensive preprocessing for feature extraction with appropriate data resampling methods to effectively address class imbalance and improve minority class classification. The choice of resampling method should consider the classification technique used to maximize benefits [23]. In addition, studies emphasize the importance of using appropriate oversampling approaches tailored to the unique characteristics of EEG datasets. For instance, the work of Lee et al. [33] showed that different oversampling methods can lead to different performance results in classification tasks, suggesting that data balancing techniques such as SMOTE can be critical in improving the predictability of brain-computer interfaces (BCIs) that rely on EEG signals. Furthermore, Fan et al. [34] identified the biases introduced into ML models through imbalanced datasets and presented a comprehensive review of oversampling methods, suggesting that these techniques are essential for improving predictive performance in various domains, including EEG classification [34].

### 3.2. Random undersampling

**Figure 2.**
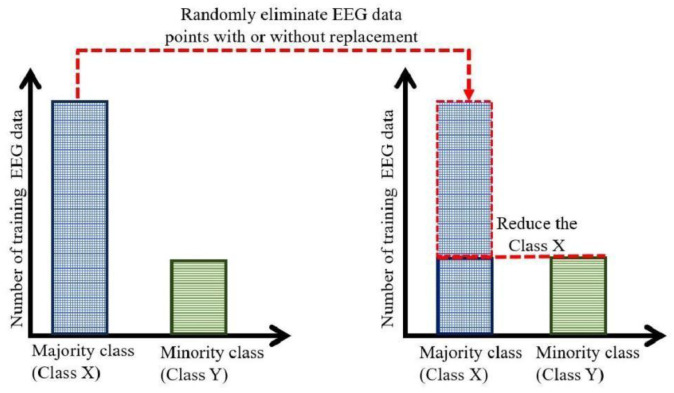
Random undersampling process of an imbalanced EEG dataset.

RUS is primarily defined as a method that indiscriminately removes instances from the majority class to achieve improved class balance [35],[36]. This undersampling technique reduces the prevalence of more frequently occurring classes, creating a more balanced dataset for training classification models. The process involves randomly selecting and eliminating examples from the majority class to reduce the bias that classification models often display toward overrepresented classes (see Figure 2). The theoretical basis of RUS is that class imbalance can cause classification models to be biased toward the majority class, reducing performance on minority class instances. RUS aims to increase classifier sensitivity to minority class instances and improve overall classification performance in imbalanced situations by reducing the prevalence of the majority class through random removal [15],[35]–[37].

Random undersampling boosting (RUSBoost) is a hybrid model that combines RUS with the AdaBoostM1 (Adaptive Boosting M1) algorithm. RUS randomly removes data points from the majority class, while AdaBoostM1 is a boosting technique used as an ensemble method to address imbalanced classes effectively by reshaping the distribution of classes before training classifiers [37],[38]. Such solutions are crucial in the management of EEG datasets, which often exhibit class imbalance due to the inherent characteristics of the recorded signals; certain events or circumstances, such as sleep spindles or outcomes of TBI, occur infrequently. The studies suggested that the use of RUSBoost in detecting abnormal EEG features exhibited a considerable enhancement in predictive capabilities, with marked improvements in metrics like classification accuracy and F1 scores compared to its counterparts, including standard AdaBoost methodologies [37],[39]. A major drawback is the potential loss of valuable data from the majority class, as the indiscriminate removal of instances can inadvertently discard informative patterns that are relevant to the classification task [40]. This problem is particularly pronounced when working with EEG data, where the subtleties of brain activity may be captured in discarded measurements. Consequently, alternative strategies, including selective undersampling that targets noisy or redundant instances, have been proposed to mitigate this loss [41].

Selective undersampling, including Tomek's Link and Edited Nearest Neighbor (ENN), have emerged as sophisticated approaches that go beyond simple RUS by strategically removing instances near the decision boundary to enhance class separation and improve classifier performance [42],[43]. In contrast to RUS techniques, which may unintentionally discard informative instances, these selected methods focus on removing noisy examples, borderline cases, and overlapping samples that contribute to classification uncertainty. Tomek's Link is a modification of the condensed nearest neighbors (CNN) algorithm refer to a specific category of instance pairs that identify potentially problematic samples near the class boundary. A Tomek Link exists when two examples from different classes are each other's nearest neighbors, meaning no other instance in the dataset is closer to either of them. Formally, two examples, *x*_*i* and *x*_*j*, from different classes form a Tomek's Link if there is no instance *x*_*k*, such that *d*(*x*_*i*, *x*_*k*) < *d*(*x*_*i*, *x*_*j*) *or* *d*(*x*_*j*, *x*_*k*) < *d*(*x*_*j*, *x*_*j*), where d is the distance function.

The CNN algorithm aims to produce a training set consistency from the original dataset that can accurately classify data points present in the original data. The primary function of Tomek Links is to eliminate overlapping samples from different classes, thereby improving class separation and lowering ambiguity [42]. In contrast, the ENN is an undersampling technique that removes a sample if it is misclassified by a nearest neighbor classifier. The ENN method primarily targets misclassified majority class samples, improving classification performance by eliminating these problematic cases. This method is highly effective because it uses the k-nearest neighbors (KNN) algorithm to discard samples near the boundary or those that are misclassified. Tomek Links and ENN require calculating distances between instances, which can be resource-intensive for large datasets. However, when implemented properly, these methods can provide computational simplicity that scales to large datasets. It is important to evaluate the trade-off between computational cost and improvement in classification when selecting appropriate undersampling methods for specific applications.

Ensemble approaches with undersampling have gained popularity as they improve performance by exploiting the diversity of trained models. Guo et al. [44] explored how methods such as UnderBagging and EasyEnsemble use RUS to generate heterogeneous classifiers and improve performance on imbalanced datasets. These methods aim to develop a diverse set of classifiers using undersampling to create balanced data subsets, thereby improving model performance by accurately representing the complexity of minority class cases. The rationale is that ensemble approaches, when used with RUS, can effectively reduce the potential information loss caused by indiscriminately eliminating majority class instances, thus enhancing classification accuracy. UnderBagging is a hybrid method that combines bootstrap aggregation (bagging) with RUS to address class imbalance. This approach randomly reduces the number of majority class instances in each bootstrap sample to match the number of minority class instances. This process creates several balanced training subsets, each containing all minority class instances and different random samples of the majority class. The main idea of UnderBagging is that training multiple classifiers on these variably balanced subsets enables the ensemble to capture diverse decision boundaries while ensuring the minority class is represented in all base learners [45].

EasyEnsemble is a collection of AdaBoost learners that are trained on differently balanced bootstrap samples, where the balancing is realized through random undersampling. This method was designed to address the class imbalance problem by creating multiple balanced subsets and training separate AdaBoost classifiers on each subset. The final prediction is obtained by combining the outputs of all AdaBoost learners, thus consolidating the information from all aspects of the unbalanced data. Although UnderBagging and EasyEnsemble use random undersampling to create balanced training subsets, they differ fundamentally in their base learner architecture and combination methods. UnderBagging typically uses homogeneous base classifiers trained with the standard bagging approach, where each classifier receives an undersampled bootstrap sample. Conversely, EasyEnsemble uses AdaBoost as its base learning algorithm, adding an additional layer of boosting within each balanced subset [45],[46]. Jiang et al. [47] highlighted a method that combines reinforcement learning with RUS to improve model resilience and point out the potential synergistic benefits of fusing RUS with advanced learning approaches. These results suggest that ensemble methods can improve the effectiveness of RUS and mitigate certain drawbacks associated with big data loss [47]. This strategy aims to improve the decision boundaries of the classifier by eliminating noisy data points, as opposed to RUS, which indiscriminately discards most instances [48]. This specificity can significantly improve classification accuracy, particularly in applications such as EEG classification, where precise decision boundaries are essential for interpretation and diagnosis [49].

## 4. EEG data augmentation techniques

Data augmentation shares conceptual similarities with traditional sampling methods such as SMOTE and RUS but offers distinct advantages by producing diverse and realistic synthetic examples. The generation of synthetic datasets increases the volume of training data, particularly for augmenting underrepresented classes. This integration shows that data augmentation and conventional sampling methods can be used together to more effectively address class imbalance than either strategy alone. Employing data augmentation involves using raw EEG data represented as a 2D matrix with dimensions *M* × *K*, where the rows correspond to channel data and the columns represent data recorded at each sampling point to generate time-frequency representations that effectively capture the EEG frequency bands. George et al. [50] highlighted six common approaches for EEG data augmentation to synthesize motor imagery signals, particularly for deep learning techniques. These techniques include trial averaging (AVG), recombination of time slices, recombination of frequency slices, Gaussian noise addition, cropping, and variational autoencoder (VAE) data synthesis [50]. The AVG for EEG data augmentation is a basic procedure in which a new synthetic trial is created by averaging several existing real trials. A specific number of real trials, referred to as N, are randomly selected from the dataset. These selected trials are then averaged to produce a single new trial. Despite varying the number of trials N selected for averaging, researchers have observed no substantial increase in decoding performance when using different numbers of epochs. This suggests that simply averaging more trials or training for longer periods does not necessarily lead to better results with this augmentation technique [50].

The process of augmenting EEG data through the random recombination of time slices involves generating new trials by randomly selected N trials and recombining roughly equal time slices from the selected trials. ensuring that the fragments retain comparable lengths. The slicing is performed in a way that respects the temporal characteristics of the EEG data, enabling the preservation of signal integrity. The segments from the selected trials are then shuffled and randomly reassembled to generate a new EEG trial. This approach aims to create distinct new trials that incorporate patterns from the original trials, thereby enhancing the dataset with diverse signal attributes [51]. Recombination of frequency slices operates within the frequency domain of EEG data to generate new samples. Moreover, EEG signals are transformed using techniques such as the Fast Fourier Transform (FFT) or Short-Time Fourier Transform (STFT) to obtain a frequency-domain representation. This enables manipulation of specific frequency bands by extracting slices corresponding to particular frequency ranges (e.g., delta, theta, alpha, beta, and gamma bands). The extracted frequency slices from different epochs or trials are then recombined to create new frequency spectra. This approach can generate novel signals that capture varied neural activity while preserving the statistical properties of the original data.

In the noise addition approach, random Gaussian noise is generated based on the statistical properties of the original EEG signal. The noise level can be controlled to ensure it does not overwhelm the genuine signal while providing variability in the training data. This generated noise is then added to randomly selected trials to create artificial frames. This simple method retains the original waveform characteristics while introducing slightly different numerical values. It is particularly beneficial in scenarios where EEG signals are naturally noisy due to artifacts or environmental factors. Moreover, cropping in EEG data augmentation enhances the diversity of training datasets, particularly when original samples are limited. The cropping process typically involves selecting time intervals that represent specific cognitive tasks or states of interest. Cropping can be combined with window slicing, in which segments of consistent length (e.g., 1–3 seconds) are extracted. This technique divides long EEG trials into smaller windows that can be analyzed by individual ML models, effectively increasing the dataset size without requiring additional recordings. For testing, prediction scores are generated from crops of a test trial, averaged, and the trial is assigned to the class with the highest mean score. This method not only generates more training instances but also helps learn task-specific patterns within a time window.

Autoencoders are a self-supervised learning framework in which the output during training approximates the input. Autoencoders typically consist of three components: The Encoder, which generates a compressed latent space representation of the input data, the Latent Space, which retains the essential information of the input data in reduced dimensionality, and the Decoder, which reconstructs the input data from the compressed latent space. Kingma and Welling [52] introduced Variational Autoencoders (VAEs) as frameworks for learning deep latent-variable models. These networks have been employed for EEG signal augmentation by capturing variations in temporal and frequency domains. VAEs are a class of generative models that provide a powerful approach to augmenting data EEG signals. A conditional variational autoencoder was used to learn the distribution of the data and to generate artificial trials based on real ones. Moreover, VAE [53] consists of two major components: Encoder and decoder. Through self-supervised learning, VAE learned to extract the effective components of EEG signals and transform them into latent representations. It can acquire the latent representations of EEG signals and generate new data samples. The latent space learned by the VAE is characterized by continuity and coherence, enabling efficient compression of EEG data and extraction of relevant information. VAEs have also been utilized to handle class imbalance issues in EEG datasets by generating synthetic samples for underrepresented classes, enhancing the overall robustness of classification models.

In a study by Sadegh-Zadeh et al. [54], VAEs were used as a data augmentation strategy to address class imbalance in the EEG dataset, particularly for minority classes, such as Mild Cognitive Impairment (MCI) patients. By generating additional samples for these underrepresented classes, VAEs helped balance the dataset, which is crucial for improving classification accuracy and stability. A VAE is a feed-forward neural network that encodes raw data into a low-dimensional vector representation and then reconstructs these vectors into artificial data. Unlike a standard autoencoder, a VAE ensures that the generated data follows a specific probability distribution by incorporating constraints into its structure. The process involves an encoder that compresses input data and a decoder that reconstructs the key properties of the data. The VAE learns that the data distribution and simulated trials are generated based on actual trials using a conditional VAE. Convolutional layers form the VAE's architecture, and oversampling the initial training set leads to better loss values and improved training stability.

### 4.1. Generative adversarial networks for EEG analysis

GANs have become powerful tools for data augmentation due to their capability to generate realistic synthetic data without explicitly modeling the probability density function [55]. GANs have been used effectively in the field of image generation [56] and have demonstrated remarkable capabilities in generating data that closely resembles real recordings, which has required rigorous examination of quantitative metrics (e.g., Fréchet inception distance (FID), structural similarity index measure (SSIM), peak to signal noise ratio (PSNR), and Euclidean distance) and qualitative metrics (e.g., visual Turing tests and neural response-based evaluation) for evaluating the quality of GAN-generated data in domains such as medical imaging and bio signals [57]. GANs consist of two major components: A generator network and a discriminator network. The generator network is trained using random vectors or values to produce synthetic data samples that exhibit structures and characteristics comparable to those of the original data. In contrast, the discriminator network is trained using both the original dataset and the generated synthetic samples. Its objective is to distinguish real data and generated (fraudulent) samples. The relationship between these two networks forms a minimax game.

**Figure 3.**
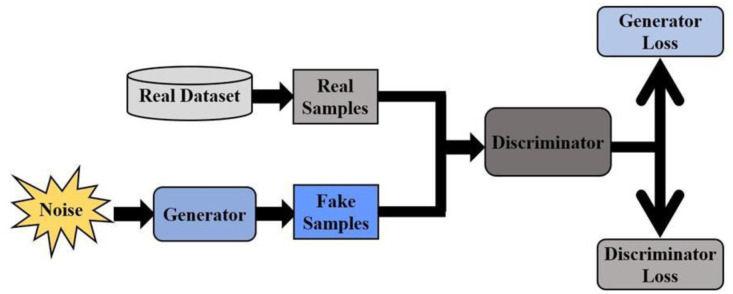
Generative adversarial network architecture.

During training, the generator aims to produce data that closely resembles genuine samples to deceive the discriminator, while the discriminator simultaneously learns to differentiate real and generated data as shown in Figure 3. The relationship between these two networks forms a minimax game. This ongoing feedback loop drives the generator to produce increasingly convincing fakes and pushes the discriminator to improve its detection abilities. Ideally, this adversarial process continues until the generator produces samples indistinguishable from real data, and the discriminator's accuracy in telling real from fake approaches 50%, indicating it can no longer differentiate them.

Beyond image generation, GANs have been increasingly applied to biomedical signal processing, particularly for EEG data augmentation, where data scarcity poses a major challenge. Augmentation of EEG data by synthetic means, particularly through the use of GANs, is a promising approach to increase the size and diversity of available training datasets [17],[58],[59]. The EEG data generated by GANs accurately replicate the statistical properties of EEG signals and specific feature representations. The synthetic EEG signals must maintain similar statistical distributions, including mean, variance, and higher-order statistics, when compared to real EEG data. This ensures that any observational patterns inherent in the real data are preserved in the synthetic data [60]. This process enables the development of more robust ML models that enable better generalization through a larger number of training examples [61]. Research shows that GAN-based models can generate EEG signals that replicate the statistical characteristics and structure of authentic EEG recordings, thereby improving the quality of datasets for training ML models [18],[62]. These results corroborate Lotte's [63] findings, which indicated that synthetic EEG trials produced by segmentation and recombination (S&R) in the temporal and frequency domains yielded more compelling outcomes. GANs use adversarial training to replicate complex rhythmic patterns typical of EEG data across several frequency bands.

Implementations of GAN-based methods have proven successful in EEG applications, including the prediction of epileptic seizures and the detection of emotions [17],[64]. For instance, research has shown that GANs can effectively generate synthetic EEG signals that are indistinguishable from real EEG recordings and can enhance models developed for specific tasks, such as brain-computer interfaces (BCI), where data scarcity and subject-specific variability pose significant challenges [65]. The effectiveness of signal indistinguishability was evaluated using quantitative metrics such as signal-to-noise ratio (SNR), PSNR, correlation coefficient, mutual information, and dynamic time warping (DTW) distance. These metrics collectively provide a comprehensive evaluation of the efficacy of GAN-based models in generating EEG signals that are indistinguishable from real clean recordings, balancing rigorous noise suppression with high-fidelity signal reconstruction. The potential of specially tailored GAN architectures, such as the combination of long short-term memory (LSTM) networks with convolutional layers, has opened ways to capture the temporal dynamics of EEG signals more effectively [58]. Nevertheless, obstacles remain, particularly regarding the need for increased realism in the EEG samples produced. Some studies suggest that while GANs can generate EEG data, the quality and authenticity of these samples need to be improved for better suitability in practical applications [66],[67]. The lack of fine-tuning in generative models may lead to the emergence of artifacts that are not present in authentic EEG data [59]. There is evidence of effective denoising strategies using GANs, where models systematically learn to detect and remove noise in EEG datasets, improving the quality of data accessible for analysis [68],[69].

Despite these successes, a major limitation of early GANs was the well-known instability of the discriminator during training. This instability can lead to mode collapse, where the discriminator recognizes only a few narrow modes of input distribution as real, causing the generator to produce a limited range of outputs. GANs may fail to converge due to unstable training dynamics arising from the interaction between the generator and discriminator. If one model becomes significantly stronger than the other, it can cause oscillatory behavior or continuous fluctuations in loss values, resulting in poor model performance. GAN training is highly dependent on architecture and hyperparameters. Different choices of learning rates, batch sizes, loss functions, and model architectures can yield markedly different results. This sensitivity poses challenges for practitioners, who may need to adjust multiple parameters without fully understanding their effects on training [17]. The efficiency of model training has been significantly improved by dedicated hardware such as GPUs, which can accelerate training times by factors of 10 to 20 [70]. However, it remains uncertain whether the time savings justify the added complexity of enhancing models for minimal performance gains. For example, methodologies aimed at faster model training, as discussed by Xu et al. [71], show that GAN training is often resource-intensive, making it difficult to decide whether slight performance improvements are worth the high computational costs [71].

The real-world application demands of deep learning models and GANs also affect the value of potential performance improvements. For example, in medical imaging, where GANs are used to generate synthetic data that enhances the performance of convolutional neural networks (CNNs) in liver lesion classification, the time spent developing these models leads to better healthcare outcomes [72]. In such cases, even a small performance increase can have significant implications for diagnostic accuracy. Additionally, this study shows that augmenting training datasets with synthetic examples generated by GANs can improve classifier performance, demonstrating that in critical applications like medicine, the processing time required for even minor performance gains may be justified. This underscores that in highly impactful fields, model efficacy outweighs computational overhead.

## 5. Review of resampling and generative data augmentation techniques in EEG-based classification

In this section, I present a review of the literature based on recent contributions to resampling and generative data augmentation techniques for dealing with imbalanced EEG datasets. Table 1 summarizes representative studies that use data augmentation techniques for EEG-based classification across application domains, including epileptic seizure detection, emotion recognition, sleep stage classification, and brain–computer interface (BCI) tasks. The studies are grouped by the data augmentation strategy: Conventional resampling-based methods (such as SMOTE variants, NearMiss, random under-sampling, or over-sampling), generative model–based approaches (e.g., GAN, WGAN, TGAN, and VAE), and hybrid or task-specific augmentation frameworks. Conventional resampling methods and generative model–based approaches are represented, with augmentation primarily used to address class imbalance.

### 5.1. Data augmentation for EEG-based seizure classification

In epileptic seizure classification, data augmentation is primarily used to address severe class imbalance, as seizure event are much less frequent than interictal or preictal states. Researchers in this field commonly use resampling techniques, such as SMOTE variants, ADASYN, NearMiss, and clusterbased oversampling methods, often combined with classical ML classifiers such as Random Forest (RF) and Support Vector Machines (SVMs). These approaches generally report improved classification performance after augmentation, especially in terms of sensitivity and F1-score for the minority seizure classes. Hu et al. [73] proposed a patient-specific framework for unbalanced preictal classification and seizure detection using multichannel EEG data. Statistical and entropy features derived from wavelet packet decomposition (WPD) were extracted, and k-means SMOTE was used to address class imbalance across epileptic states. However, the authors noted that the effectiveness of the approach was limited by the short duration and limited availability of original seizure events, raising questions about its generalizability to high-imbalanced real clinical data. The enlarged training set produced by k-means SMOTE significantly increased training time, which may hinder rapid model updates or deployment in time-sensitive clinical settings, despite the relatively efficient inference phase.

Alhudhaif [74] showed that the integration of the ADASYN and the RF classifier achieved the highest success rate in classifying EEG signals into five class datasets containing various EEG signals, including epilepsy datasets, reaching an accuracy of 91.72%. Compared to baseline RF and decomposition-based methods (OVA and OVO), the ADASYN-integrated framework significantly improved classification results by alleviating class imbalance in the five-class EEG dataset. The augmentation method enhanced the identification of epileptic EEG patterns, underscoring the importance of adaptive oversampling when minority classes are underrepresented. This method, combining OVA + ADASYN + RF, achieved the highest classification success rate of 91.72% for the five-class EEG signals, outperforming the other three approaches presented in the study. This approach is recognized for its effectiveness in detecting epileptic occurrences in EEG recordings.

Masum et al. [75] systematically evaluated undersampling, oversampling, and hybrid resampling techniques to address class imbalance in epileptic seizure detection. This work showed that the integration of NearMiss undersampling with a Support Vector Machine (SVM) classifier yielded optimal performance for identifying epileptic seizures from imbalanced datasets, achieving a beta score of 0.996, precision of 0.999, and recall of 0.989, outperforming traditional ML and deep learning models. This result highlights the importance of selective undersampling in preserving informative majority-class samples while improving minority-class discrimination. However, the authors noted that undersampling methods such as NearMiss may discard potentially valuable EEG data, which could limit generalizability in highly heterogeneous clinical datasets. Additionally, although deep neural networks (DNN) demonstrated competitive performance, their higher computational requirements and sensitivity to data imbalance pose practical challenges for deployment in real-world clinical settings.

Abou-Abbas et al. [76] systematically evaluated DA approaches for enhancing classification performance in seizure detection. This included comparing GAN variants, such as Wasserstein Generative Adversarial Network with Gradient Penalty (WGAN-GP), Vanilla GAN, Conditional GAN (CGAN), and Cramer GAN, against traditional oversampling methods like SMOTE and ADASYN. The results showed that WGAN-GP was the most effective GAN variant for DA when used with RF classifiers, exhibiting a remarkable resemblance to real data, unlike other GAN variants that failed to capture inherent patterns. Incorporating synthetic data through augmentation significantly improved the overall performance of the RF classification model. The accuracy increased from 0.86 (real data only) to 0.88 (real + synthetic data). Projection analysis and Q-Q plots demonstrated that WGAN-GP generated synthetic data closely resembled real data distributions, indicating its ability to capture inherent patterns and structures. In contrast, Vanilla GAN and CGAN failed to capture these patterns effectively, showing significant deviation from real data. CramerGAN showed some improvement over Vanilla GAN and CGAN, but WGAN-GP exhibited superior performance in generating realistic synthetic data. The authors further integrated the WGAN-GP with bidirectional long short-term memory (BiLSTM) networks, which yielded substantial performance improvement in seizure detection. This combined approach achieved an accuracy of 91.73% on augmented data, significantly outperforming the 86% accuracy achieved for real data without augmentation. WGANGP also demonstrated the lowest loss value (0.0681) and the highest precision (80.81%) and recall (76.71%) among the tested data augmentation approaches for LSTM. With WGAN-GP, the imbalanced datasets in epilepsy when seizure events were rare compared to non-seizure events were successfully addressed. The researchers' successful application of WGAN-GP for data augmentation directly addressed this challenge by generating synthetic minority class data that closely resembled real data, thereby balancing the dataset and improving model performance. By achieving a high accuracy of 91.73% on augmented data, the proposed method offers a more reliable tool for automatic epileptic seizure detection. This enhanced accuracy means fewer missed seizures (higher recall) and fewer false alarms (higher specificity), which are critical for effective patient care and management.

In a relevant study, Ganti et al. [77] emphasized that even limited seizure datasets can benefit from data augmentation methods, and they found an improvement in classification accuracy of seizure episodes through GAN implementations. They proposed using temporal GAN (TGAN) to produce synthetic data to increase the sample size of thalamic SEEG data. This synthetic data should improve the efficiency of BiLSTM classifiers, which are designed to distinguish ictal (seizure) and interictal (ground state) states. BiLSTM models are adept at extracting patterns from temporal data and preserving dependencies across longer time sequences. The synthetic data generated by TGAN significantly improved the performance of BiLSTM in classifying thalamic ictal and baseline states. The accuracy of the recognition model increased by 18.5% with the inclusion of the synthetic data.

Additional metrics showed significant improvement: AUC increased from 60% (original data) to 78.5% (synthetic data), and sensitivity and positive predictive value (PPV) increased by 13% and 10%, respectively, for the test data. A significant difficulty for GAN models, including TGAN, is the risk of generating erroneous data while processing discontinuous datasets, such as electrocorticography (ECoG) acquired from therapeutic neuromodulation devices. This indicates that the model may encounter difficulties with sudden alterations or irregular patterns in the input data. The study provided proof of-concept, emphasizing enhanced accuracy with TGAN augmentation despite a limited sample size. Nevertheless, more thorough validation is required, encompassing random sampling of patient data and validation at the individual subject level.

Kan et al. [78] used Gramian-based matrix transformations in conjunction with ResNet topologies to improve the classification of multichannel EEG data. Their methodology aimed to introduce an innovative technique to synthesize multichannel EEG as Gramian Angular Field (GAF) images through a newly developed Gramian Temporal Generative Adversarial Network (GT-GAN), which facilitated the generation of synthetic GAF images that improved the accuracy of EEG anomaly detection within residual learning frameworks. Thus, enriching the datasets with authentic synthetic data can increase the efficiency of anomaly detection algorithms. A major goal is to ensure that the proposed technique accurately represents the temporal dependencies found in EEG recordings through visual representations. The use of GAF images aims to preserve the spatial and temporal properties of the original EEG data during the transformation process. This addresses a shortcoming of previous EEG synthesis techniques that did not sufficiently account for these temporal correlations. The developed GT-GAN has shown to produce realistic GAF images. In contrast to other EEG synthesis methods, this paradigm considers the temporal relationships between EEG signals. Experimental results have shown that incorporating the images generated by GT-GAN into the training dataset significantly increases the classification accuracy of the ResNet models (ResNet-20, ResNet56, and ResNet-110) using the Temple University Hospital Abnormal EEG Corpus (TUAB) dataset. The ResNet-110 model trained with the augmented dataset achieved a classification error of 13.5%, a significant reduction from its original error rate of 29.0%. Similarly, the error rate of ResNet-20 decreased from 44.2% to 34.8%, while the error rate of ResNet-56 dropped from 39.9% to 21.4%. The classification error of ResNet-110 on the augmented dataset was found to be worse than all other proposed classification systems by Shah et al. [79]. Moreover, by enhancing the accuracy of EEG anomaly detection, the GT-GAN framework can lead to more reliable and earlier identification of neurological conditions. This is particularly relevant for conditions where early diagnosis can significantly impact patient outcomes. The result shows that the proposed method, even using only the first 10 seconds of EEG recordings, effectively preserves information for seizure detection [78].

Despite apparent methodological diversity, the reviewed studies collectively demonstrate several consistent findings regarding data augmentation in epileptic seizure classification. Across these studies, classical resampling techniques (e.g., SMOTE variants, ADASYN, and NearMiss) and advanced generative models (e.g., WGAN-GP and TGAN) effectively mitigate class imbalance and improve detection of minority seizure classes. Notably, performance gains are observed consistently, with reported improvements ranging from modest accuracy increases of approximately 2% using WGANGP with RF classifiers to substantial gains of up to 18.5% when TGAN is combined with BiLSTM models, along with marked improvements in precision and recall. The choice between classical resampling and generative augmentation reflects trade-offs related to computational cost, synthetic data fidelity, and generalizability, rather than fundamental differences in their ability to enhance classification performance. The heterogeneity in reported evaluation metrics (accuracy, precision, recall, F1-score, and AUC) primarily reflects variations in dataset characteristics, EEG acquisition modalities (scalp EEG, SEEG, and ECoG), and classifier architectures, rather than intrinsic incomparability between studies. When considered within their respective experimental contexts, the findings converge on a coherent narrative: Data augmentation substantially enhances seizure detection performance. In this regard, generative models, particularly WGAN-GP, demonstrate superior capability in producing realistic synthetic EEG signals, whereas classical resampling methods offer greater computational efficiency and more established validation frameworks. Nevertheless, a shared limitation across all approaches is the challenge of validating the physiological authenticity of augmented data and ensuring robust generalizability to diverse clinical populations with heterogeneous seizure phenotypes and recording conditions.

### 5.2. Data augmentation for EEG-based emotion recognition

In recent EEG-based emotion recognition research, Wardoyo et al. [80] addressed class imbalance in the Dataset for Emotion Analysis using EEG and Physiological and Video Signals Database for Emotion Analysis using Physiological Signals (DEAP)by combining differential entropy (DE) feature extraction with the Radius-SMOTE oversampling strategy. DE is a widely used EEG representation for emotion recognition that summarizes signal statistics in a compact feature form and is frequently used to build learning-ready feature matrices for classifiers. To address imbalance, Wardoyo et al. [80] used Radius-SMOTE, an extension of SMOTE designed to reduce overlap and noise amplification that can occur when synthesizing minority samples in standard oversampling schemes. Specifically, Radius-SMOTE introduces a radius-based selection and filtering mechanism to identify “safe” minority instances and constrain synthetic-sample generation within a controlled neighborhood, limiting the creation of ambiguous samples near class boundaries and reducing overlap with other class regions. This design directly addresses known limitations of conventional oversampling approaches, where naive replication or unconstrained interpolation can increase overfitting or blur decision boundaries by regulating where and from which minority instance synthetic points are produced. Therefore, integrating Radius-SMOTE with DE-based EEG features is presented as a principled way to improve the reliability of training data for downstream classifiers in DEAP-style arousal/valence recognition settings.

Zhang et al. [81] presented an Emotional Subspace Constrained Generative Adversarial Network (ESC-GAN) developed to improve the representation of emotional states derived from EEG data by modifying reference EEG signals. This breakthrough highlights the model's ability to navigate between well-represented and under-represented emotional subspaces, achieving a fairer representation of different emotional expressions in EEG data. A unique editing paradigm is provided that transforms reference EEG signals from well-represented emotional categories to under-represented emotional subspaces using two innovative loss functions. The diversity-focused loss function promotes a diverse emotional subspace by improving sample differentiation and thus preventing the amplified EEG signals from becoming too homogeneous. This facilitates the inclusion of underrepresented emotions to establish an unbiased decision boundary. Boundary-aware loss restricts the extended subspace near the decision boundary, where recognition models exhibit increased vulnerability. This helps in the defense against antagonistic attacks. It is designed to increase resilience against potential attacks on underrepresented emotions and guarantees accurate classification of augmented samples near the decision boundary. Moreover, experiments show that ESC-GAN significantly improves the efficiency of emotion identification in benchmark datasets such as DEAP, AMIGOS, and SEED. The strategy outperforms other GAN-based data augmentation techniques and leading algorithms. The proposed method also shows advantages in resisting attacks by attackers, suggesting that it can create a more resilient classification boundary. This is especially important considering that adversarial attacks are more severe on imbalanced datasets.

The analyzed studies collectively indicate that data augmentation is an effective strategy for addressing the class imbalance in EEG-based emotion recognition, particularly for arousal and valence classification tasks. Both controlled resampling approaches, such as Radius-SMOTE, and constraintaware generative models, such as ESC-GAN, consistently improve classification performance by enhancing the representation of underrepresented emotional states. The effectiveness of RadiusSMOTE depends strongly on the quality of the underlying differential entropy features and the appropriateness of the selected radius parameter for the given dataset and feature space. However, this resampling strategy lacks an explicit mechanism for validating the physiological plausibility or clinical relevance of the generated samples, relying primarily on geometric proximity to real data as a surrogate measure of quality. While resampling-based methods are computationally efficient and straightforward to implement, generative approaches offer greater flexibility in reshaping emotional subspaces and improving robustness near decision boundaries. In particular, ESC-GAN's demonstrated resistance to adversarial attacks, along with its improved recognition of underrepresented emotions, addressing a critical reliability gap in EEG-based emotion recognition systems. By positioning augmented samples in feature-space regions that are inherently more robust to perturbations, ESC-GAN reduces the risk of misclassification arising from natural signal variability or adversarial manipulation. This capability is especially relevant for safety-critical applications, such as mental health monitoring and affective human–computer interaction. However, these advantages come with increased model complexity and training requirements. Overall, these findings underscore that effective data augmentation for EEGbased emotion recognition requires not only balancing class distributions but also carefully managing sample variability and decision-boundary behavior to preserve physiological plausibility, class separability, and robust generalization across subjects and datasets.

### 5.3. Data augmentation for EEG-based sleep stage classification

Krishnamoorthy [82] addressed the class imbalance problem in sleep phase models by duplicating minority class samples. Training the CNN on imbalanced data without specific mitigation strategies resulted in a strong bias toward the majority class, yielding high specificity but low sensitivity; in other words, the model accurately identified non-microsleep states but performed poorly in detecting actual microsleeps. In contrast, Fan et al. [24] demonstrated that data augmentation methods, primarily using GANs, significantly improved overall classification performance compared to baseline models for sleep stage classification. Krishnamoorthy [82] also compared simple oversampling of the minority class (microsleep states) with a cost-based learning approach using weighted cross-entropy to address imbalanced CNN training data. The findings reaffirmed that training on unbalanced datasets without appropriate handling leads to majority-class bias and suboptimal sensitivity. The study also highlighted practical challenges associated with oversampling raw EEG data, as generating synthetic EEG epochs was not feasible due to memory constraints and the high dimensionality of the data. As a result, repeated replication of minority class samples required splitting the training data into multiple batches, which substantially increased training time and computational burden. Given these limitations, cost based learning offers a computationally efficient alternative to data-level imbalance handling. This approach uses weighted cross-entropy loss functions during model training. Instead of modifying the training data, it assigns different weights to predict errors based on class membership, applying higher penalties to misclassifications of the minority class. By incorporating class weights directly into the loss function, cost-based learning reduces the amount of data processed and shortens training time compared to oversampling-based methods.

Thus, it is demonstrated that training on unbalanced datasets without appropriate management can lead to bias in favor of the majority class, resulting in high specificity but low sensitivity. It provides practical insights into the difficulties of oversampling raw EEG data, as generating synthetic EEG epochs for minority classes is difficult. EEG recordings contain complex temporal patterns reflecting underlying neural processes, and traditional oversampling techniques often fail to preserve these critical characteristics (e.g., ensuring adequate signal-to noise ratios for EEG data) when generating synthetic data. High SNR is required to maintain the physiological integrity of synthetic EEG data, as brain signals contain subtle but important patterns that can be obscured or distorted by excessive noise. Generating synthetic EEG data must preserve the delicate balance between signal and noise that characterizes authentic brain recordings, ensuring that meaningful neural activity is distinguishable from background electrical activity and recording artifacts.

In addition, it was found that memory limitations caused by oversampling required splitting the replication training data into batches, which increased the training time. This problem was mitigated by cost-based learning. The performance metrics for oversampling and cost-based learning techniques were very similar in detecting microsleep states. This suggests that both methods can be effective in overcoming data imbalances in terms of detection accuracy. The cost-based learning method had a significantly shorter training time compared to the oversampling method. Oversampling the minority class sometimes caused the training data to exceed the memory requirements of the GPU, which required splitting into multiple batches and repeated training sessions, which was time consuming. In contrast, by applying appropriate weights during prediction error calculation, cost-based learning reduces the amount of data to be processed and thus shortens the training time.

Fan et al. [24] found that data augmentation techniques, especially those using generative adversarial networks (DAGAN), significantly improve the overall classification efficiency of sleep disorder categorization. This improvement was seen in the classification measures of accuracy, the F1 score, and Cohen's kappa coefficient (κ) for the Montreal Archive of Sleep Studies (MASS) and Sleep-EDP datasets. In the study, the DAGAN consistently showed superior performance compared to the other four data augmentation (DA) methods, which included minority class repetition (DAR), EEG signal morphological change (DAMC), signal segmentation and recombination (DASR), and dataset-to-dataset transfer (DAT). In the MASS dataset, DAGAN led to improvements of 3.79% in accuracy, 3.48% in F1 score and 5.43% in κ. It also showed improvements of 4.51% in accuracy, 3.14% in F1 score, and 5.8% in κ in the Sleep-EDF dataset. The F1 scores achieved by DAGAN differed significantly from baseline in the MASS and Sleep-EDF datasets (p < 0.05). This represents a substantial and statistically significant improvement in classification performance. The exceptional performance of DAGAN in classifying the sleep stages highly depended on the architecture of the network. In DAGAN, the convolutional neural networks (CNNs) function as the primary classifier for evaluating the impact of data augmentation on sleep classification accuracy and as essential components within the GAN architecture for generating synthetic EEG data. CNN layers are essential to the design of the GANs formulated in this study. Moreover, the generator and discriminator components of the GAN models integrate CNN layers. The generator uses upsampling blocks with CNN layers to augment input vectors, while the discriminator uses downsampling techniques implemented by CNN layers to reduce the dimensions. The integration of CNN layers with activation functions such as Leaky ReLU and local response normalization enables the GAN to generate and discriminate authentic and synthetic EEG signals.

### 5.4. Data augmentation for EEG-based BCI classification

Fahimi et al. [62] discussed a framework utilizing deep convolutional GANs (DCGANs) to generate synthetic EEG data and to bolster the dataset utilized for training BCI classifiers. This method enhances the robustness of the classifier, proving that GAN-based augmentation can lead to improved performance without the need for extensive new data collection. Specifically, the proposed DCGANs-based augmentation led to a significant improvement of 7.32% in accuracy for diverted attention conditions and 5.45% for focused attention conditions (both with p < 0.01). This indicated that the synthetic samples generated by DCGANs were more effective in contributing new, useful information to the training set, leading to better classification performance. A more recent generative approach, the diffusion-based augmentation model [83]–[85], has gained attention for EEG analysis. It operates through a forward diffusion process that learns to generate new samples by removing noise. Torma and Szegletes [84] demonstrated that diffusion models can effectively create high-quality EEG samples, representing significant progress toward efficient and generalizable augmentation techniques for diverse EEG decoding tasks. The forward diffusion process gradually degrades data by adding Gaussian noise over multiple timesteps, while the reverse process learns to denoise and generate new samples that match the original distribution. Moreover, incorporating diffusion-based augmentation directly into training pipelines enables more sophisticated data enhancement approaches that can adapt to specific model requirements and training dynamics. This integration represents a significant advantage over traditional augmentation methods, which typically operate as preprocessing steps separate from model training. Despite the promising results of EEG synthetic data generation [85], diffusion-based augmentation involves significant computational considerations, particularly regarding the trade-off between sample quality, the number of sampling steps, and generation speed [84],[85].

The proposed DCGANs for generating artificial EEG data were compared against two other generative methods, which were VAE and S&R. While VAE is recognized as a powerful generative method, the study revealed that the proposed conditional DCGANs significantly outperformed VAE, especially in the diverted attention condition. In terms of accuracy, VAE did not achieve a significant improvement over the baseline in either focused or diverted conditions when used for augmentation. Specifically, in the diverted condition, VAE achieved the highest true-positive rate (TPR) at 85.71% but had the lowest specificity at 67.14% among the methods. The S&R method is divided into time domain S&R and time-frequency domain S&R. These methods involve dividing EEG trials into segments and then concatenating random segments (time domain) or their short-time frequency transform (STFT) window (time-frequency domain) to generate artificial samples. Similar to VAE, the S&R methods did not lead to a significant improvement in classification accuracy over the baseline in either the focused or diverted conditions when used for data augmentation. The authors emphasized that leveraging such a data generation method is time-and cost-efficient, which resonates well within the often logistically constrained settings of EEG data collection [62]. The practical applications of GANs in EEG data augmentation extend beyond mere quantitative improvements.

A significant improvement in GAN applications is also evident in the research of Du et al. [58], who presented an extended GAN model developed for the augmentation of EEG signal data. Their approach emphasized the need for temporal integrity of EEG signals to ensure that the synthetically generated data accurately reflects real brain processes. Du et al. [58] integrated the Wasserstein-GAN with gradient penalty (WGAN-GP) and used the Wasserstein distance as a loss function to improve the stability and efficiency of training. The generator element of the L-C-WGANGP model was created using LSTM networks. Moreover, the LSTM was selected due to its ability to capture long-term dependencies in sequential data, preserve distant contextual information, and learn the temporal attributes of EEG signal limitations [56]. The discriminator consists of a CNN. In contrast to the generator, it does not have a forgetting unit or recurrent connections, which enables faster training with extensive sequence data. This combination takes advantage of deep learning to analyze the statistical features of EEG signals and generate synthetic data that closely resembles the actual samples.

The L-C-WGAN-GP model extends training datasets by generating new EEG data. This data augmented approach improves the size and diversity of the training data and, in combination with the gradient penalty-based Wasserstein distance as a loss function, increases the performance and robustness of deep learning models. Compared to other GANs, including DCGAN, WGAN, WGANGP, and LSTM-GAN, L-C-WGAN-GP consistently achieves the lowest root mean square (RMSE), Fréchet distance (FD), and dynamic time warping (WTD) values for evaluating the quality and similarity of synthetic data. The L-C-WGAN-GP model consistently shows superior performance on all similarity assessment metrics (RMSE, FD, and WTD). The L-C-WGAN-GP model consistently achieves the lowest scores for RMSE, FD, and WTD compared to all other models. This indicates that the EEG data generated by L-C-WGAN-GP is very similar to authentic EEG data, demonstrating its improved ability to assimilate the attributes of EEG signals. Thus, its RMSE was 0.21, its FD was 0.75 and its WTD was 10.38, which are the optimal values among all models. DCGAN had the highest values for all three-similarity metrics (RMSE: 0.71, FD: 0.99, WTD: 23.71). This indicates that DCGAN generated the poorest quality EEG data among the models compared. The main reason for this poor performance is its susceptibility to gradient vanishing during training, which hinders the ability of the generator to effectively learn the basic features of the EEG data.

WGAN showed better performance compared to DCGAN, as reflected by a lower RMSE (0.40), FD (0.92), and WTD (16.89). This improvement is attributed to the application of Wasserstein distance, which mitigates certain training issues associated with conventional GANs. WGAN-GP improved WGAN and achieved a lower RMSE (0.37), FD (0.89), and WTD (15.23). The inclusion of a gradient penalty in WGAN-GP mitigates the gradient explosion, increases the training speed, and stabilizes the training process, resulting in better data quality. LSTM-GAN, which uses LSTM networks for the generator and discriminator, showed competitive results with RMSE (0.26), FD (0.81), and WTD (12.87). Despite its commendable performance, it was outperformed by the proposed L-CWGAN-GP model. The L-C-WGAN-GP model outperformed other leading GAN models by utilizing an LSTM-based generator to capture temporal dependencies and a CNN-based discriminator for effective discrimination, along with the stability provided by the WGAN-GP loss function. This exceptional performance, as evidenced by reduced similarity metrics and visually enhanced signal features, confirms the effectiveness of the model in generating high-quality artificial EEG data that closely resembles authentic samples. Despite this progress, it is essential to address persistent problems. Although GANs can address the imbalance between classes, the samples generated must remain faithful to the real signals to prevent the introduction of noise or deceptive artifacts that could affect future studies. A trend is emerging in the research landscape where the integration of GANs with sophisticated deep learning approaches such as LSTMs is seen as an effective approach to mitigate analog deficiencies [56].

In this research, CNNs are used in the compressed sensory reconstruction model for EEG inputs. Incorporating the generated EEG data into the original dataset for training significantly improves the accuracy of compressed sensory reconstruction (CSR), and CNNs are one of the models used for this purpose. For CNN reconstruction, using 25% to 100% of the generated data improved the average reconstruction accuracy by 0.06% to 0.17% compared to using the original material only, especially at compression rates between 40% and 90%. At a compression level of 30%, the improvement was between 0.33% and 1.22%. Similarly, the accuracy of the CS-ResNet reconstruction increased by an average of 0.05% to 0.14% by including the supplemented data. At a compression rate of 30%, the improvement was between 0.3% and 0.80%. At lower compression rates (e.g. 10% and 20%), notable improvements were seen, with reconstruction accuracy increasing by 1.51% to 5.58% with CNN and by 0.87% to 4.69% with CS-ResNet when the augmented data were included. The authors conclude that their proposed L-C-WGAN-GP model efficiently generates approximations for EEG data that, when used for enhancement, improve the performance and stability of EEG signal reconstruction models by mitigating overfitting. Notably, Habashi et al. [17] provides a comprehensive overview of the applications of GANs in EEG analysis, highlighting the effectiveness of these networks for augmenting limited training data while reducing dependency on prolonged calibration sessions. The authors suggest that GAN-generated samples can adhere closely to the distribution characteristics of original EEG signals, thus ensuring that the newly introduced data points are not only numerically sufficient but also quality-compatible with existing datasets. This notion is further explored in research by Du et al. (2024) who describe the use of GANs to generate augmented EEG signals while maintaining the distribution characteristics of the original data [17].

### 5.5. Class imbalance in TBI EEG datasets for prognostication

Despite the clinical promise of EEG-based TBI prognosis, a critical methodological challenge remains largely unaddressed in the literature: The class imbalance problem inherent in TBI outcome datasets. TBI outcome distributions are typically skewed, with favorable outcomes occurring more frequently than unfavorable outcomes in many patient populations [6]. This fundamental characteristic of TBI epidemiology creates substantial challenges for ML models trained on imbalanced datasets, as models tend to develop bias toward the majority class, resulting in high specificity but critically low sensitivity for minority class detection [15].

Noor et al. [15] explicitly acknowledged the class imbalance problem in TBI outcome prediction and proposed using RUSBoosted Trees on electroencephalography spectral power to improve TBI outcome prediction from unbalanced datasets. This work is one of the few to explicitly recognize class imbalance as a serious methodological problem in EEG-based TBI prognostication. However, the proposed solution relies on classic ensemble methods rather than modern data augmentation techniques. The authors noted that, given the limitations of current prognostic biomarkers, there is growing interest in understanding the role of new biomarkers derived from quantitative EEG features based on ML algorithms. While GAN-based data augmentation has not been widely used in TBI prognosis, strong evidence demonstrates the usefulness of these approaches in related EEG applications, particularly seizure detection [86]. A critical research gap exists at the intersection of two well-established methodological domains: (1) GAN-based data augmentation for addressing imbalanced EEG datasets and (2) ML approaches for TBI outcome prediction. Although both domains have made substantial progress independently, their integration is notably absent from the published literature.

On the other hand, ML approaches for TBI outcome prediction have been actively developed, with multiple researchers proposing deep learning architectures for EEG-based prognostication [87],[88]. However, these researchers have not systematically incorporated advanced data augmentation techniques, particularly GAN-based approaches, to address the inherent class imbalance in TBI outcome datasets.

Yang et al. [89] identified the need for more valuable observation indicators to improve the accuracy of early prognosis prediction in TBI patients. Moreover, models based on comprehensive factors and parameters from medical history, physical examination, and supplementary examinations could provide an accurate and reliable prediction of the early prognosis of TBI patients. However, the authors noted that other biomarkers, such as EEG, computed tomography perfusion (CTP), and cerebral oxygen saturation, which may be related to TBI, should be appropriately incorporated into prediction models, as this increases the predictive value of these models in predicting prognosis [90]. Notably, this recommendation does not address the methodological challenge of handling imbalanced datasets, suggesting that the class imbalance problem may not have been adequately recognized as a critical limitation in TBI prognostic modeling.

The lack of generative augmentation approaches (e.g., GAN-based) in TBI EEG prognosis has important clinical implications. The failure to incorporate GAN-based data augmentation represents a missed opportunity to leverage established techniques for improving model performance. Moreover, accurate outcome prediction is crucial for clinical decision-making, especially in severe TBI cases where prognostic uncertainty can result in suboptimal treatment choices. If the class imbalance problem is not addressed, models may have high specificity but low sensitivity for minority outcome classes, potentially causing missed opportunities for intervention in patients with an unfavorable prognosis. In future studies, researchers should systematically explore the adaptation of established GAN architectures to EEG-TBI prognostication, with particular attention to preserving physiological validity, integrating multimodal prognostic indicators, and evaluating performance across TBI severity levels. The successful integration of GAN-based data augmentation in other EEG-based applications has the potential to substantially improve prognostic accuracy and clinical relevance.

However, the introduction of GAN-based data augmentation and other increasingly complex deep learning architectures also raises important cost–benefit considerations, particularly in EEG-based clinical applications. More complex models are inherently more difficult to maintain, debug, and modify, especially when unexpected failures occur or when models must be adapted to evolving data distributions or clinical requirements. The operational costs associated with increased complexity, including longer processing times, higher power consumption, greater memory requirements, reduced interpretability, and increased maintenance burden, may outweigh marginal gains in predictive performance. Rather than prioritizing increasingly complex models, researchers studying EEG should focus on developing efficient architectures that achieve strong performance within the constraints of real-world clinical environments. This shift requires a reassessment of research priorities, emphasizing optimization of the cost–benefit tradeoff and the establishment of standardized evaluation frameworks that consider computational efficiency and deployability alongside traditional performance metrics.

**Table 1.** Summary of the works leveraging data augmentation (DA) techniques for EEG-based classification.

Application	Resampling Technique		Classifiers	Model performance evaluation metrics					Limitations	Ref.
Epileptic seizure classification	k-mean SMOTE	Blending with k-mean SMOTE	CHB-MIT database performance (Average accuracy %)						The effectiveness of the proposed approach is dependent on the quality and relevance of these hand-crafted features. The imbalance of the data is directly addressed by k-means SMOTE. Nevertheless, the main problem remains is the duration of seizure onset (ictal state) is much shorter than that of preictal and interictal states. k-means SMOTE helps to balance the training data set. Nevertheless, the lack of real seizures in the real world remains an obstacle to generalizability to new, highly imbalanced data.	Hu et al. [73]
			Original database						
			RF	Mean Acc (%)	Mean F1 (%)				
				78.10	72.97				
			Blending	89.49	85.81				
			Resampled with k-mean SMOTE						
			RF	Mean Acc (%)	Mean F1 (%)				
				97.66	97.23				
			Blending	98.08	97.74				
			iNeuro database performance (Average accuracy %)						
			Original database						
			RF	Mean Acc (%)	Mean F1 (%)				
				61.64	73.19				
			Blending	69.50	77.34				
			Resampled with k-mean SMOTE						
			RF	Mean Acc (%)	Mean F1 (%)				
				-	-				
			Blending	92.68	80.93				
Epileptic seizure classification	k-mean SMOTE	Random forest	Method used	Classification accuracy (%)					By synthesizing new data point, ADASYN can alter the original data distribution and potentially shift or blur the decision boundaries between classes. While the goal is to improve the classifier's ability to learn the minority class, if not carefully controlled, this could lead to misclassification of majority class instances or a less robust overall model, especially in complex, high-dimensional data like EEG signals.	Alhudhaif [74]
				RF with raw EEG	64.80					
				125 time-domain features + RF	71.90					
				One-Versus-All (OVA) + RF	89.00				
				One-Versus-One (OVO) + RF	91.08				
				One-Versus-All (OVA) + RF + ADASYN	91.72				
	NearMiss + SVM	SVM	Method used	F-β	PPV		Recall		The study primarily examines a range of traditional and state-of-the-art sampling methods (e.g. SMOTE, ADASYN, NearMiss, SMOTEENN, SMOTETomek) and machine learning classification methods (SVM, LR, RF, DT, DNN). Although comprehensive in scope, it does not explore all possible equalization methods or advanced deep learning architectures that might exist.	Masum et al. [75]
			NearMiss + SVM	0.996	0.999		0.989		
			NearMiss + DNN	0.990	0.997		0.973		
			ROS + SVM	0.971	0.969		0.945		
			ROS + DNN	0.979	0.972		0.967		
			RUS + SVM	0.962	0.965		0.921		
			RUS + DNN	0.961	0.958		0.925		
			NearMiss + SVM	0.996	0.999		0.989		
			Cluster Centroids + SVM	0.946	0.954		0.882		
			Cluster Centroids + DNN	0.952	0.953		0.900		
			ADASYN + SVM	0.963	0.955		0.937		
			ADASYN + DNN	0.970	0.956		0.957		
			SMOTEEN + SVM	0.977	0.974		0.950		
			SMOTEEN + DNN	0.958	0.978		0.973		
			SMOTETTomek + SVM	0.977	0.974		0.950		
	TGAN	BiLSTM	Original data	PPV	SN	SP	F1	AUC	The TGAN model is computationally intensive and requires substantial time to learn and converge to local minima. This characteristic significantly slows down the training process, making it resource demanding.	Ganti et al. [77]
				62	60	59	60	60		
			TGAN-Augmented data	72	73	63	72	78.5		
	WGAN-GP	CNN	Classification Error Rates of Models Before and After GT-GAN Enhancement						The study focused on only P3-01, T5-01, and F7-T3) and used only the first 10 seconds of EEG recordings for GAF matrix creation. It might not capture all relevant information present in longer or multi- channel EEG recordings, potentially limiting the generalizability of the findings to more complex EEG analysis tasks.	Kan et al. [78]
			Model	Original Error Rtae (%)			Enhanced Error Rate (%)		
			ResNet-20	44.2			34.8		
			ResNet-56	39.9			21.4		
			ResNet-110	29.0			13.5		
	WGAN-GP	BiLSTM, RF	Method	Precision	Recall	Specificity	Accuracy	F1-score	The study underscores the necessity for more research and evaluation to determine the generalizability of its findings across various datasets. The present work offers significant insights. Nonetheless, its conclusions are based on a particular dataset (Temple University Hospital EEG Seizure Corpus), and performance may differ with alternative epileptic datasets.	Abou-Abbas et al. [76]
			Real data only	0.81	0.79	0.67	0.86	0.83		
			Real + Synthetic data	0.84	0.83	0.72	0.88	0.86		
			SMOTE	0.1252	0.603	0.705	0.889	0.8525		
			ADASYN	0.1281	0.6150	0.7191	0.8847	0.8518		
			Class Weight	0.1255	0.6038	0.7293	0.876	0.8482		
Emotional State Recognition	ESC-GAN		Comparative Performance on Benchmark Datasets	Arousal Accuracy (%)			Valence Accuracy (%)		ESC-GAN leverages an adjustable adversarial loss to generate realistic samples that enhance classification performance and robustness for underrepresented emotions. However, this design introduces a trade-off, as the focus on classification support may limit the consistency of sample realism compared with GANs optimized purely for distribution approximation.	Zhang et al. [81]
			DEAP Dataset	96.33%			96.68 %			
			AMIGOS Dataset	94.40%			95.23%			
			SEED Dataset	97.14%						
	Radius SMOTE	CNN, Decision Trees	Without Resampling	Model	Arousal Acc (%)		Valence Acc (%)		Notwithstanding the advancements realized by Radius-SMOTE, the overall accuracy of emotion identification for both arousal and valence emotions persists below 85%. This signifies that there remains potential for enhancement in the precision of emotion identification derived from EEG signals.	Wardoyo et al. [80]
				DT	63.86		62.52			
				CNN	69.55		68.56			
			Radius-SMOTE	DT	78.78		75.14			
				CNN	82.11		78.99			
	DAGAN	CNN	Dataset	Acc	F1		κ		The GAN-based DA (DAGAN) method, despite its superior performance, is significantly more complex and resource-intensive than other approaches. Training the GAN models consumed approximately 71.69 hours, with an additional 19.63 minutes for artificial signal generation	Fan et al. [24]
			Montreal Archive Sleep Study (MASS)	0.767 ± 0.009	0.692 ± 0.018		0.656 ± 0.018		
			Sleep-EDF	0.748 ± 0.023	0.685 ± 0.021		0.660 ± 0.023		
	Oversampling, Cost-sensitive based learning	CNN		AUCPR	Phi		AUCROC		Limited full exploration of CNN including experimenting with different hyperparameters, layers and CNN structures (sequential/parallel) to optimize performance. Customizing layers to reduce inter-subject variability. This indicates that the current CNN model might not generalize well across different individuals without further modification. Absent of ensemble techniques limit the model performance. Incorporation of ensemble techniques, which might have yielded better detection capabilities of microsleeps	Krishnamoorthy [82]
			Oversampled	0.41	0.42		0.90		
			Cost-based learning	0.40	0.40		0.90		
Brain Computer Interface	L-C-WGANGP		Model	Performance Metrics					High computational load of LSTM. The LSTM component within the model, particularly in the generator, incurs significant computational costs during its operation. Limited generalizability for different EEG datasets. The current focus of this study uses EEG signals data from motor imagination as training dataset. The model generalizability to other EEG datasets or different EEG signals (e.g., for emotion recognition, epilepsy prediction) has not completely been validated. Future work is planned to generalize the proposed method to more datasets.	Du et al. [58]
				RMSE	FD		DTW			
			DCGAN	0.71	0.40		0.37			
			WGAN	0.99	0.92		0.89			
			WGAN-GP	23.71	16.089		15.23			
			LSTM-GAN	0.71	0.40		0.37			
			L-C-WGAN-GP (Proposed Model)	0.99	0.92		0.89			
	DCGANs	CNN	Method	Condition: Focused (Mean ± SD)			Condition: Diverted (Mean ± S D)		The study highlights the need for a more comprehensive analysis of how the generated samples influence the features learned by the classifier. Understanding the meaningfulness of these changes is crucial for validating the augmentation process and could provide deeper insights into why GAN-based augmentation improved classification, especially under diverted attention conditions	Fahimi et al. [62]
			VAE	81.19 ± 8.75			76.43 ± 8.58			
			S&R in time domain	81.26 ± 7.85			74.04 ± 8.55			
			S&R in time-frequency domain	81.53 ± 7.69			74.04 ± 8.55			
			Conditional DCGANs	85.54 ± 7.36			80.36 ± 7.46			

Note: Acc = accuracy; ADASYN = adaptive synthetic sampling approach; AUC = area under curve; BiLSTM = bidirectional long short-term memory; DA = data augmentation; DAGAN = data augmentation based on generative adversarial network; DCGANs = deep convolutional generative adversarial networks; DNN = deep neural network; DTW = dynamic time warping; ESC-GAN = emotional subspace constrained generative adversarial network; FD = Fréchet distance; GMM-HMM = Gaussian mixture model–hidden Markov model; HMM-SdA = hidden Markov model–stacked denoising autoencoder; LSTM = long short-term memory; PCA-HMM = principal component analysis–hidden Markov model; RMSE = root mean square error; ROS = random oversampling; RUS = random undersampling; SMOTE = synthetic minority oversampling technique; SVM = support vector machine; WGAN-GP = Wasserstein GAN with gradient penalty.

## 6. Conclusions

In summary, sampling methods can improve the prediction of underrepresented classes in binary and multi-class classification problems, which are common in EEG-based classification tasks for detecting conditions such as TBI. In binary settings, random undersampling reduces majority-class bias by removing redundant samples, but this may lead to the loss of potentially informative data. In contrast, oversampling techniques increase minority-class representation but may cause overfitting by generating duplicate or highly similar samples that do not fully capture the intrinsic variability of EEG signals. More advanced deep learning–based balancing strategies, particularly GANs, have shown greater robustness and improved classification accuracy in binary and multi-class EEG paradigms. Moreover, training classifiers on balanced datasets that adequately represent all outcome categories can enhance predictive performance and support better prognostic assessment in TBI and related neurological disorders. In future studies, researchers should investigate the integration of complementary GAN architectures to further improve class balancing and advance the clinical applicability of EEG-based analysis frameworks.

## Use of AI tools declaration

The authors declare they have not used Artificial Intelligence (AI) tools in the creation of this article.
